# Impact of recombinant human brain natriuretic peptide on emergency dialysis and prognosis in end-stage renal disease patients with type 4 cardiorenal syndrome

**DOI:** 10.1038/s41598-023-48125-1

**Published:** 2023-11-25

**Authors:** Yue Zhou, Xiaojian Wang, Hongbo Yuan, Linke Wu, Bin Zhang, Xiaoxia Chen, Yafeng Zhang

**Affiliations:** 1 Department of Nephrology, Nanjing First Hospital, Nanjing Medical University, Nanjing, 210006 China; 2 Department of Nephrology, BenQ Medical Center, The Affiliated BenQ Hospital of Nanjing Medical University, Nanjing, 210019 China; 3 Department of Respiratory, Nanjing First Hospital, Nanjing Medical University, Nanjing, 210006 China; 4 Department of Cardiology, Nanjing Yuhua Hospital, Nanjing, 210039 China; 5 Department of Public Health, Affiliated Hospital of Jiangsu University, Zhenjiang, 212003 China

**Keywords:** Drug discovery, Immunology, Diseases

## Abstract

Recombinant human brain natriuretic peptide (rhBNP) effects on type 4 cardiorenal syndrome (CRS) and adverse events such as heart failure rehospitalization and all-cause mortality have not been assessed in large-scale research. This study evaluated the impact of rhBNP on emergency dialysis and prognosis in end-stage renal disease (ESRD) patients with type 4 CRS, and the risk factors of emergency dialysis. This retrospective cohort study included patients with type 4 CRS and ESRD admitted for decompensated heart failure between January 2016 and December 2021. Patients were divided into the rhBNP and non-rhBNP cohorts, according to whether they were prescribed rhBNP. The primary outcomes were emergency dialysis at first admission and cardiovascular events within a month after discharge. A total of 77 patients were included in the rhBNP cohort (49 males and 28 females, median age 67) and 79 in the non-rhBNP cohort (47 males and 32 females, median age 68). After adjusting for age, residual renal function, and primary diseases, Cox regression analysis showed that rhBNP was associated with emergency dialysis (HR = 0.633, 95% CI 0.420–0.953) and cardiovascular events (HR = 0.410, 95% CI 0.159–0.958). In addition, multivariate logistic regression analysis showed that estimated glomerular filtration rate (eGFR) (OR = 0.782, 95% CI 0.667–0.917, P = 0.002) and procalcitonin (PCT) levels (OR = 1.788, 95% CI 1.193–2.680, P = 0.005) at the first visit were independent risk factors for emergency dialysis while using rhBNP was a protective factor for emergency dialysis (OR = 0.195, 95% CI 0.084–0.451, P < 0.001). This study suggests that RhBNP can improve cardiac function and reduce the occurrence of emergency dialysis and cardiovascular events in ESRD patients with type 4 CRS.

## Introduction

Type 4 cardiorenal syndrome (CRS) is a medical condition in which chronic kidney disease leads to heart injury, disease, and/or dysfunction^1^. The incidence of cardiovascular events increases with the deterioration of renal function^2^. Moreover, it has been reported that patients with CKD stage 5 are 4–5 times more likely to succumb to cardiac arrest than patients with lower CKD stages^3,4^. Even in the maintenance dialysis stage, CKD patients have a high incidence of cardiovascular events and a cardiogenic sudden death rate (7–8 folds higher) than the general population^4^. Therefore, delaying premature entry into the dialysis phase and reducing the cardiovascular events of type 4 CRS is vitally important.

Brain natriuretic peptide (BNP) is a component of the natriuretic peptide (NP) system mainly secreted by cardiac myocytes that has an important role in cardiorenal protection^5^. During heart failure, increased ventricular load and wall tension lead to activation of the *BNP* gene in the cardiomyocytes, resulting in the overproduction of the BNP protein that regulates vasodilation, diuresis, and sodium excretion^5^. Yet, in the stage of decompensated heart failure, endogenous BNP is insufficient to compensate for the deterioration of cardiac function^6^; thus, exogenous BNP supplementation may be recommended to alleviate the rapidly embittered hemodynamic status and improve heart function^6^.

Human recombinant human brain natriuretic peptide (rhBNP), also known as nesiritide, has already been approved by the Federal Drug Administration (FDA) for acute decompensated congestive heart failure (CHF) in the USA since 2001^7^. In China, rhBNP has been used to treat acutely decompensated heart failure with dyspnea at rest or minimal activity. rhBNP has the same amino acid sequence, spatial structure, and biological activity as endogenous BNP and can attenuate myocardial fibrosis, inhibit cardiac remodeling, improve inflammation, and regulate vasodilating and antagonize aldosterone without adverse effects on renal function^8,9^. Yet, no large-scale research has assessed its effects on type 4 CRS, especially whether it can delay dialysis and reduce adverse events such as heart failure rehospitalization and all-cause mortality. Therefore, this study evaluated the impact of rhBNP on emergency dialysis, prognosis in end-stage renal disease (ESRD) patients with type 4 CRS, and the risk factors of emergency dialysis.

## Methods

### Study design and population

This retrospective cohort study included ESRD patients with type 4 CRS admitted to Nanjing First hospital for decompensated heart failure between January 2016 and December 2021. The inclusion criteria were: (1) patients who met the diagnostic criteria for type 4 CRS defined by the expert consensus published by Kidney Disease: Improving Global Outcomes (KDIGO) and Acute Dialysis Quality Initiative group (ADQI) in 2010^1^ with eGFR < 15 ml/min; (2) age ≥ 18 years of age. The exclusion criteria were: (1) patients who had received maintenance dialysis or emergency dialysis before enrollment; (2) patients without acute decompensated heart failure occurred before and after admission; (3) lost during follow-up; (4) patients who were still hospitalized at the time of this study; (5) discharged less than 6 months; (6) patients with urinary tract obstruction, severe hepatic function, renal malignancy, surgical history of heart or kidney; (7) allergies to rhBNP.

### Data collection and definitions

Patients were divided into the rhBNP and non-rhBNP cohorts, according to whether they were prescribed rhBNP (rhBNP was included in the Chinese National Reimbursement Drug List in July 2017, thereafter in our hospital it was commonly used in patients with acute decompensated heart failure, while no patients had been prescribed before). All patients received conventional treatment regimens for chronic kidney disease and heart failure, and the cases with additional rhBNP were included in rhBNP cohort. rhBNP was prescribed when patients present with symptoms of acute decompensated heart failure, and the usage referred to the medicine instruction and the payment standard of medical insurance: the loading dose 1.5–2.0 μg/kg for intravenous injection, and the maintenance dose 0.0075–0.01 μg/kg·min by continuous intravenous infusion for three days. All patients received intravenous injection of loop diuretic tolasemide according to the instrucions, at an initial dosage of 20 mg, followed by further increases up to the maximum dose (100 mg daily) indicated in the instructions if the effectiveness was unsatisfactory, with a course of treatment not exceeding one week. The vast majority of patients did not receive inotropes. Patients with unrelieved heart failure received dialysis. In case of subsequent recurrence of cardiovascular events, the rhBNP cohort was re-prescribed rhBNP if indicated.

The demographic and clinical characteristics were collected from medical records: (1) sex, age, body mass index (BMI), and primary diseases, which included diabetes, hypertension, glomerulonephritis, and other kidney diseases; (2) daily urine output and renal function indicators, including blood urea nitrogen (BUN), serum creatinine (Scr), and estimated glomerular filtration rate (eGFR) according to the chronic kidney disease epidemiology collaboration (CKD-EPI) equation^10^; (3) serum albumin(ALB), electrolytes including serum potassium(K) and sodium(Na) levels; (4) heart failure biomarkers including N-Terminal-pro-B-type natriuretic peptide (NT-proBNP) and soluble growth stimulation express gene 2 (sST2) levels; (5) inflammation indicators including high sensitivity C-reactive protein (hsCRP), interleukin-6 (IL-6), and procalcitonin (PCT) levels; (6) echocardiographic indices including left ventricular ejection fraction (LVEF), pulmonary artery systolic pressures (PASP), left ventricular end-diastolic diameter (LVDd), and left ventricular end-systolic diameter (LVDs).

D value is defined as pre-treatment–post-treatment. Percentage of improvement = (pre-treatment − post-treatment)/pre-treatment × 100%.

### Outcomes

The primary outcomes were emergency dialysis at first admission and cardiovascular events within a month after discharge; the secondary outcomes were entry to maintenance dialysis (long-term renal replacement therapy), cardiovascular events, or death within six months after discharge. Emergency dialysis was defined as hemodialysis initiated immediately when a patient presents with a life-threatening condition, including uremia with severe hyperkalemia, hypertension, or heart failure that is difficult to control with medication^11,12^. Cardiovascular events associated with renal failure include acute heart failure, cardiogenic shock, acute coronary syndrome, and related low cardiac output syndromes^13^.

### Statistical analysis

SPSS Version 24.0 (IBM Corp., Armonk, NY, USA) was used to perform statistical analyses. Continuous variables with normal distribution are described as mean and standard deviation, and then a t-test was applied. Measurement data conforming to skewed distribution are described as median and interquartile range, and a nonparametric test was used. Categorical variables are described as frequencies and proportions and compared using the Chi-square or Fisher’s exact test. In statistical analyses, factors considered potential confounders were age, residual renal function, and primary diseases^14–16^. The Cox regression analysis was used to analyze the hazard ratio of rhBNP for primary and secondary outcomes. Binary logistic regression was used to analyze the risk factors for emergency dialysis. Receiver operating characteristic (ROC) analysis was used to evaluate the performance of diagnostic tests and the accuracy of a statistical model.* P* value < 0.05 was considered statistically significant.

### Ethics approval and consent to participate

This work has been carried out in accordance with the Declaration of Helsinki (2000) of the World Medical Association. This study was approved by the ethical committee of Nanjing First Hospital [KY20220425-02]. This article is a retrospective study. Therefore Ethics Committee of Nanjing First Hospital waived the requirement to obtain distinct written informed consent from the patients.

## Results

### Baseline characteristics

**Figure 1:**
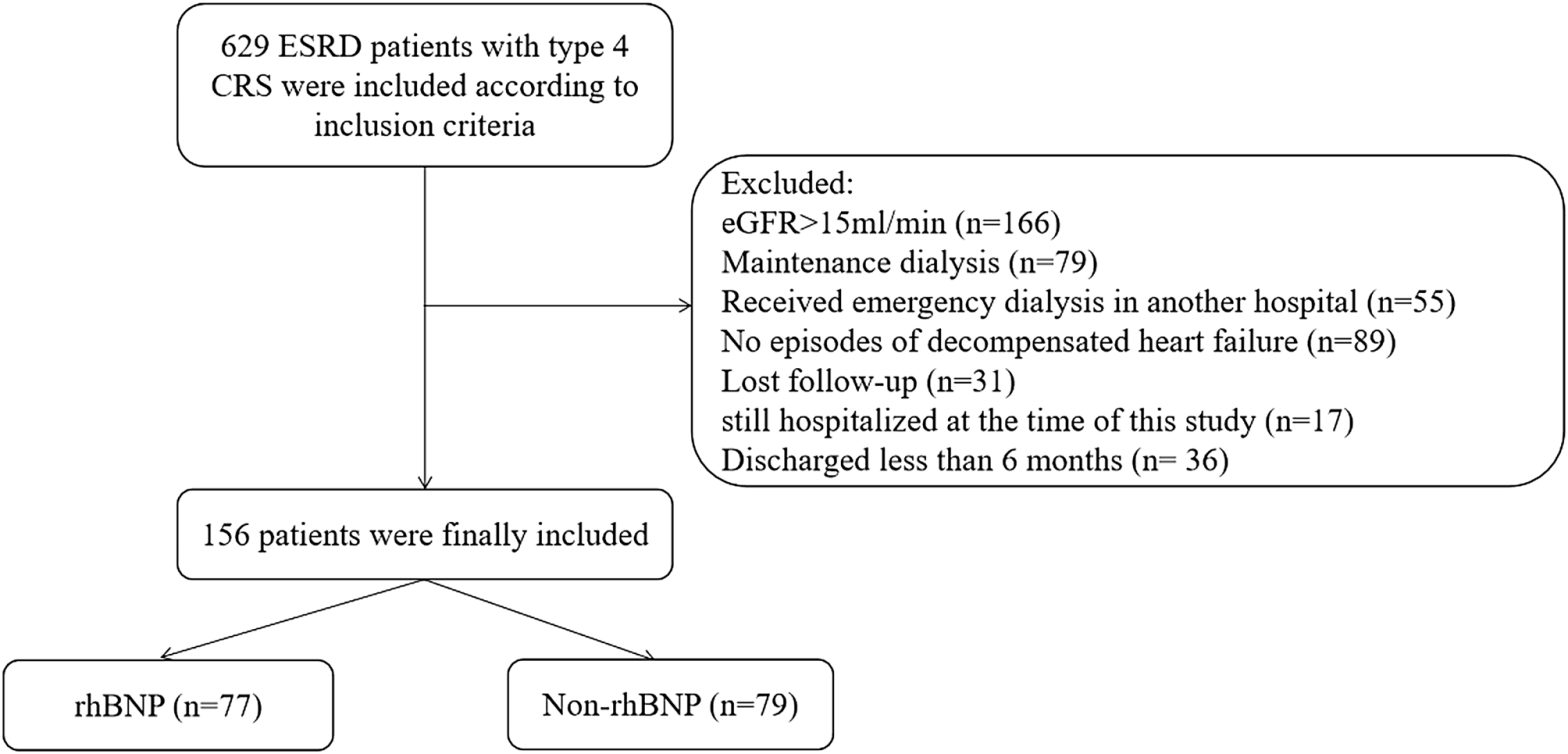
Study flowchart.

Initially, 629 patients with CRS type 4 were included. After screening patients with ESRD, with and without dialysis and with decompensated heart failure, 156 remained, 79 in the non-rhBNP cohort and 77 in the rhBNP cohort (Fig. 1). There were no significant differences in sex, age, BMI, LVEF, residual renal function (eGFR at visit), and primary diseases between the two cohorts (P > 0.05, Table 1).

**Table 1:** Baseline characteristics of the patients.

Characteristic	rhBNP cohort (n = 77)	non-rhBNP cohort (n = 79)	*p*-value
Sex, n (male/female)	49/28	47/32	0.595
Age (years)	67 (59, 77)	68 (60, 76)	0.767
BMI (kg/m^2^)	25.36 ± 4.23	25.09 ± 3.95	0.683
Primary diseases			
Diabetes, n	30	28	0.746
Glomerulonephritis, n	19	18	
Hypertension, n	15	14	
Other, n	13	19	
Daily urine output (ml)	1000 (753, 1260)	1020 (865, 1237)	0.580
ALB (g/L)	32.60 (29.18, 36.55)	32.35 (29.53, 34.93)	0.681
BUN (mmol/L)	23.45 (19.65, 31.01)	24.40 (18.32, 28.70)	0.962
Scr (μmol/L)	572.0 (475.4, 795.0)	598.8 (462.0, 784.0)	0.960
eGFR (ml/min/1.73mm^2^)	6.83 (5.11, 9.15)	6.96 (5.35, 9.10)	0.953
K (mmol/L)	4.49 ± 0.64	4.38 ± 0.61	0.258
Na (mmol/L)	141.2 (138.6, 143.3)	141.0 (138.6, 143.7)	0.947
IL-6 (pg/ml)	14.47 (9.00, 20.73)	14.87 (8.76, 22.18)	0.830
hsCRP (mg/L)	3.98 (0.72, 13.95)	4.17 (1.06, 18.76)	0.335
PCT (ng/ml)	1.73 (0.79, 2.39)	1.63 (1.01, 2.60)	0.640
NT-proBNP (pg/ml)	17,341 (12,173, 23,250)	16,989 (9704, 24,781)	0.240
sST2 (ng/ml)	42.30 (25.42, 76.83)	47.90 (30.85, 88.00)	0.359
LVEF (%)	58 (47, 63)	56 (49, 58)	0.069
PASP (mmHg)	39 (37, 53)	41 (38, 48)	0.510
LVDd (mm)	53.0 (51.0, 58.0)	52.0 (50.0, 52.5)	0.002
LVDs (mm)	36.0 (33.4, 41.3)	33.5 (32.0, 36.0)	< 0.001

*BMI* Body mass index, *ALB* albumin, *BUN* blood urea nitrogen, *Scr* serum creatinine, *eGFR* estimated glomerular filtration rate, *IL-6* interleukin-6, *hsCRP* high sensitivity C-reactive protein, *PCT* procalcitonin, *NT-proBNP* N-Terminal-pro-B-type natriuretic peptide, *sST2* soluble growth stimulation express gene 2, *LVEF* left ventricular ejection fraction, *PASP* pulmonary artery systolic pressures, *LVDd* left ventricular end-diastolic diameter, *LVDs* left ventricular end-systolic diameter.

### Parameters of the two cohorts' pre- and post-treatment

Parameters of both cohorts improved to varying degrees after treatment, including increased urine volume, improved renal function indices, decreased serum potassium, and heart failure markers (all P < 0.05). In addition, inflammatory indicators and echocardiographic parameters of the rhBNP cohort improved after treatment (all P < 0.05), while no change was seen in the non-rhBNP cohort (all P > 0.05) (Table 2). Table 3 compares the improvements of the two cohorts pre and post-treatment. Compared with the non-rhBNP cohort, the increase in the urine volume and the decrease in hsCRP and sST2 after treatment were more significant in the rhBNP cohort (all P < 0.05). The improvement of LVEF, PASP, LVDd, and LVDs in the rhBNP cohort was also greater than those of the non-rhBNP cohort (all P < 0.05).

**Table 2:** D-values of clinical parameters before and after treatment.

Parameter	rhBNP cohort (n = 77)		Non-rhBNP cohort (n = 79)	
	D-value*	*p-*value	D-value*	*p*-value
Daily urine output (ml)	− 200 (− 388, 12.50)	** < 0.001**	− 46.97 ± 295.99	0.165
ALB (g/L)	− 0.05 ± 2.71	0.893	0 (− 1.35, 2.10)	0.864
BUN (mmol/L)	2.09 (− 1.39, 7.35)	**0.005**	4.42 ± 10.85	**0.001**
Scr (μmol/L)	16.00 (− 68.75, 119.50)	0.182	67.27 ± 213.47	**0.007**
eGFR (ml/min/1.73mm^2^)	− 0.43 ± 2.61	0.158	− 0.82 ± 2.89	**0.015**
K (mmol/L)	0.23 ± 0.67	**0.006**	0.19 ± 0.73	**0.028**
Na (mmol/L)	− 0.30 (− 2.25, 1.65)	0.443	− 1.02 ± 12.55	0.484
IL-6 (pg/ml)	2.60 (− 0.81, 5.62)	**0.001**	− 0.99 ± 20.81	0.674
hsCRP (mg/L)	0.39 (− 0.82, 6.26)	**0.025**	− 0.90 (− 12.30, 9.22)	0.909
PCT (ng/ml)	0.58 ± 1.80	**0.006**	0.14 (− 0.22, 0.70)	0.172
NT-proBNP (pg/ml)	4817.00 (1931.00, 7223.54)	** < 0.001**	3339.23 (323.55, 7166.00)	** < 0.001**
sST2 (ng/ml)	8.73 (1.22, 21.95)	** < 0.001**	6.16 ± 19.23	**0.006**
LVEF (%)	− 0.79 ± 2.71	**0.014**	0.14 ± 1.51	0.809
PASP (mmHg)	2.17 ± 4.48	** < 0.001**	0.18 ± 4.66	0.743
LVDd (mm)	0.39 ± 1.64	**0.045**	0.06 ± 0.47	0.321
LVDs (mm)	0.52 ± 2.08	**0.033**	0.08 ± 0.79	0.412

*ALB* albumin, *BUN* blood urea nitrogen, *Scr* serum creatinine, *eGFR* estimated glomerular filtration rate, *IL-6* interleukin-6, *hsCRP* high sensitivity C-reactive protein, *PCT* procalcitonin, *NT-proBNP* N-Terminal-pro-B-type natriuretic peptide, *sST2* soluble growth stimulation express gene 2, *LVEF* left ventricular ejection fraction, *PASP* pulmonary artery systolic pressures, *LVDd* left ventricular end-diastolic diameter, *LVDs* left ventricular end-systolic diameter.

Significant values are in bold.

*D value = pre-treatment parameter level − post-treatment parameter level.

**Table 3:** Percentage of improvement before and after treatment*

Parameter	rhBNP cohort (%, n = 77)	Non-rhBNP cohort (%, n = 79)	t/z	*p*-value
Daily urine output (ml)	− 18.00 (− 33.00, − 4.00)	− 1.50 (− 18.75, 9.00)	− 3.280	**0.001**
ALB (g/L)	− 1.00 (− 5.00, 5.00)	0 (− 4.00, 6.75)	− 0.192	0.848
BUN (mmol/L)	8.65 ± 0.30	7.99 ± 0.42	0.111	0.911
Scr (μmol/L)	3.12 ± 0.23	4.42 ± 0.30	− 1.006	0.314
eGFR (ml/min/1.73mm^2^)	− 5.00 (− 35.00, 15.00)	− 5.50 (− 36.00, 15.00)	− 1.003	0.316
K (mmol/L)	6.00 (− 3.00, 13.00)	4.50 (− 7.75, 12.75)	− 0.336	0.737
Na (mmol/L)	− 0.21 (− 1.63, 1.17)	0 (− 1.00, 2.00)	− 1.178	0.239
IL-6 (pg/ml)	18.00 (− 7.00, 45.00)	29.00 (− 64.00, 58.75)	− 1.480	0.139
hsCRP (mg/L)	37.00 (− 50.00, 75.00)	− 90.50 (− 445.50, 63.50)	2.082	**0.037**
PCT (ng/ml)	17.00 (− 31.00, 46.00)	8.50 (− 23.25, 37.00)	1.710	0.087
NT-proBNP (pg/ml)	24.00 (11.00, 41.00)	25.50 (1.00, 37.00)	1.946	0.052
sST2 (ng/ml)	23.00 (4.00, 41.00)	12.50 (− 7.75, 31.00)	2.106	**0.035**
LVEF (%)	− 1.68 ± 0.61	0.04 ± 0.03	− 2.161	**0.032**
PASP (mmHg)	4.52 ± 0.11	0.05 ± 0.09	2.751	**0.007**
LVDd (mm)	0.84 ± 0.03	0.11 ± 0.01	2.063	**0.041**
LVDs (mm)	1.44 ± 0.05	0.22 ± 0.02	1.984	**0.049**

*ALB* albumin, *BUN* blood urea nitrogen, *Scr* serum creatinine, *eGFR* estimated glomerular filtration rate, *IL-6* interleukin-6, *hsCRP* high sensitivity C-reactive protein, *PCT* procalcitonin, *NT-proBNP* N-Terminal-pro-B-type natriuretic peptide, *sST2* soluble growth stimulation express gene 2, *LVEF* left ventricular ejection fraction, *PASP* pulmonary artery systolic pressures, *LVDd* left ventricular end-diastolic diameter, *LVDs* left ventricular end-systolic diameter.

Significant values are in bold.

*Percentage of improvement = (pre-treatment parameter level − post-treatment parameter level)/pre-treatment parameter level × 100%.

### Primary and secondary outcomes

**Figure 2:**
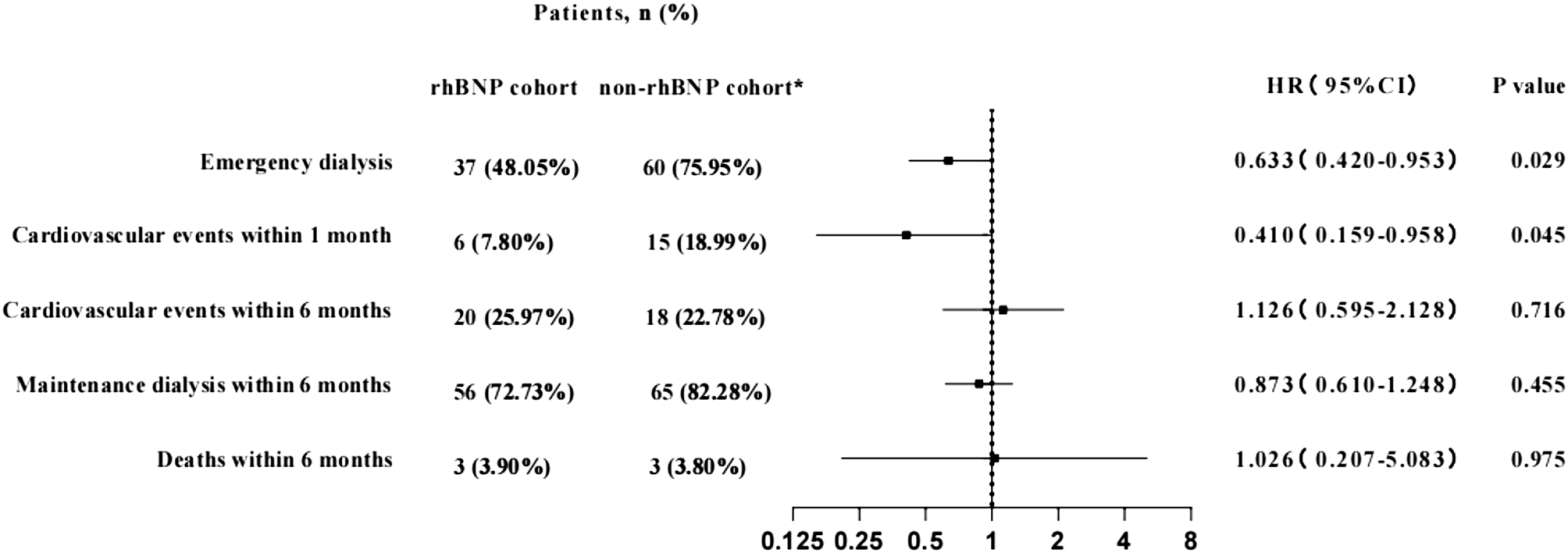
Primary and secondary outcomes in the two cohorts. Reference: Non-rhBNP cohort. *One paitent in the non-rhBNP cohort died within one month after discharge.

The number of patients with adverse events was compared between the two cohorts. The number of patients with major adverse events in the rhBNP cohort was lower than that in the non-rhBNP cohort (all P < 0.05). After adjusting age, residual renal function, and primary diseases, patients in the rhBNP cohort had 0.633 times the risk of undergoing emergency dialysis at first hospitalization (HR = 0.633, 95% CI 0.420–0.953) and 0.410 times the risk of cardiovascular events within one month (HR = 0.410, 95% CI 0.159–0.958) compared to those in the non-rhBNP cohort. One patient in the non-rhBNP cohort died within one month after discharge; the remaining 78 patients were compared for subsequent adverse events. There were no significant differences between cohorts in the risk ratios of receiving maintenance hemodialysis, cardiovascular events, and death within six months (all P > 0.05, Fig. 2).

### Risk factors for emergency dialysis

A total of 97 patients received emergency dialysis. The baseline data of all patients were analyzed by logistic regression. Univariate analysis showed that the levels of BUN, Scr, eGFR, hsCRP, PCT, and using rhBNP were associated with emergency dialysis (all P < 0.05, Table 4). After adjusting age, residual renal function, and primary diseases, the multivariate regression analysis showed that eGFR (OR = 0.782, 95% CI 0.667–0.917, P = 0.002), PCT (OR = 1.788, 95% CI 1.193–2.680, P = 0.005, while no cases of severe infection were identified in this study) at the first visit were independent risk factors for emergency dialysis and rhBNP was a protective factor for emergency dialysis (OR = 0.195, 95% CI 0.084–0.451, P < 0.001) (Table 5).

**Table 4:** Univariable analysis for emergency dialysis.

Parameter	Emergency dialysis (n = 97)	Without emergency dialysis (n = 59)	*p*-value
Age (years)	68.00 (60.00, 78.00)	67.00 (59.00, 75.00)	0.872
BMI (kg/m^2^)	25.00 ± 4.25	25.51 ± 3.84	0.457
ALB (g/L)	32.20 ± 5.64	32.88 ± 3.83	0.418
BUN (mmol/L)	25.18 (19.60, 34.30)	22.20 (18.52, 26.60)	**0.023**
Scr (μmol/L)	661.00 (482.00, 885.00)	522.30 (440.00, 619.00)	**0.001**
eGFR (ml/min/1.73mm^2^)	6.71 ± 2.65	8.30 ± 2.69	** < 0.001**
K (mmol/L)	4.44 ± 0.62	4.41 ± 0.65	0.789
Na (mmol/L)	140.90 (138.00, 143.40)	141.90 (139.00, 143.70)	0.560
IL-6 (pg/ml)	15.34 (9.98, 29.19)	14.31 (8.76, 19.14)	0.090
hsCRP (mg/L)	4.85 (1.56, 22.20)	2.23 (0.45, 10.58)	**0.003**
PCT (ng/ml)	2.01 (1.51, 2.91)	1.16 (0.37, 1.72)	** < 0.001**
NT-proBNP (pg/ml)	19,540 (11,023, 24,800)	16,735 (11,285, 19,683)	0.100
sST2 (ng/ml)	44.40 (25.51, 93.72)	41.05 (27.67, 69.90)	0.355
LVEF (%)	57 (48, 59)	58 (49, 62)	0.304
PASP (mmHg)	40 (37, 48)	40 (38, 54)	0.670
LVDd (mm)	52 (51, 53)	52 (49, 56)	0.765
LVDs (mm)	35 (33, 38)	35 (32, 40)	0.697
Primary diseases			
Diabetes, n	38	20	0.275
Glomerulonephritis, n	18	19	
Hypertension, n	19	10	
Other, n	22	10	
Using rhBNP, n			
Yes	37	40	** < 0.001**
No	60	19	

*BMI* body mass index, *ALB* albumin, *BUN* blood urea nitrogen, *Scr* serum creatinine, *eGFR* estimated glomerular filtration rate, *IL-6* interleukin-6, *hsCRP* high sensitivity C-reactive protein, *PCT* procalcitonin, *NT-proBNP* N-Terminal-pro-B-type natriuretic peptide, *sST2* soluble growth stimulation express gene 2, *LVEF* left ventricular ejection fraction, *PASP* pulmonary artery systolic pressures, *LVDd* left ventricular end-diastolic diameter, *LVDs* left ventricular end-systolic diameter.

Significant values are in bold.

**Table 5:** Multivariable conditional logistic regression analysis for emergency dialysis.

Parameter	B	SE	Wal	*p*-value	OR	95% CI
eGFR	− 0.246	0.081	9.182	0.002	0.782	0.667–0.917
PCT	0.581	0.206	7.915	0.005	1.788	1.193–2.680
rhBNP (Ref: Non-rhBNP)	− 1.635	0.428	14.616	< 0.001	0.195	0.084–0.451

*eGFR* estimated glomerular filtration rate, *PCT* procalcitonin, *rhBNP* recombinant human brain natriuretic peptide.

**Figure 3:**
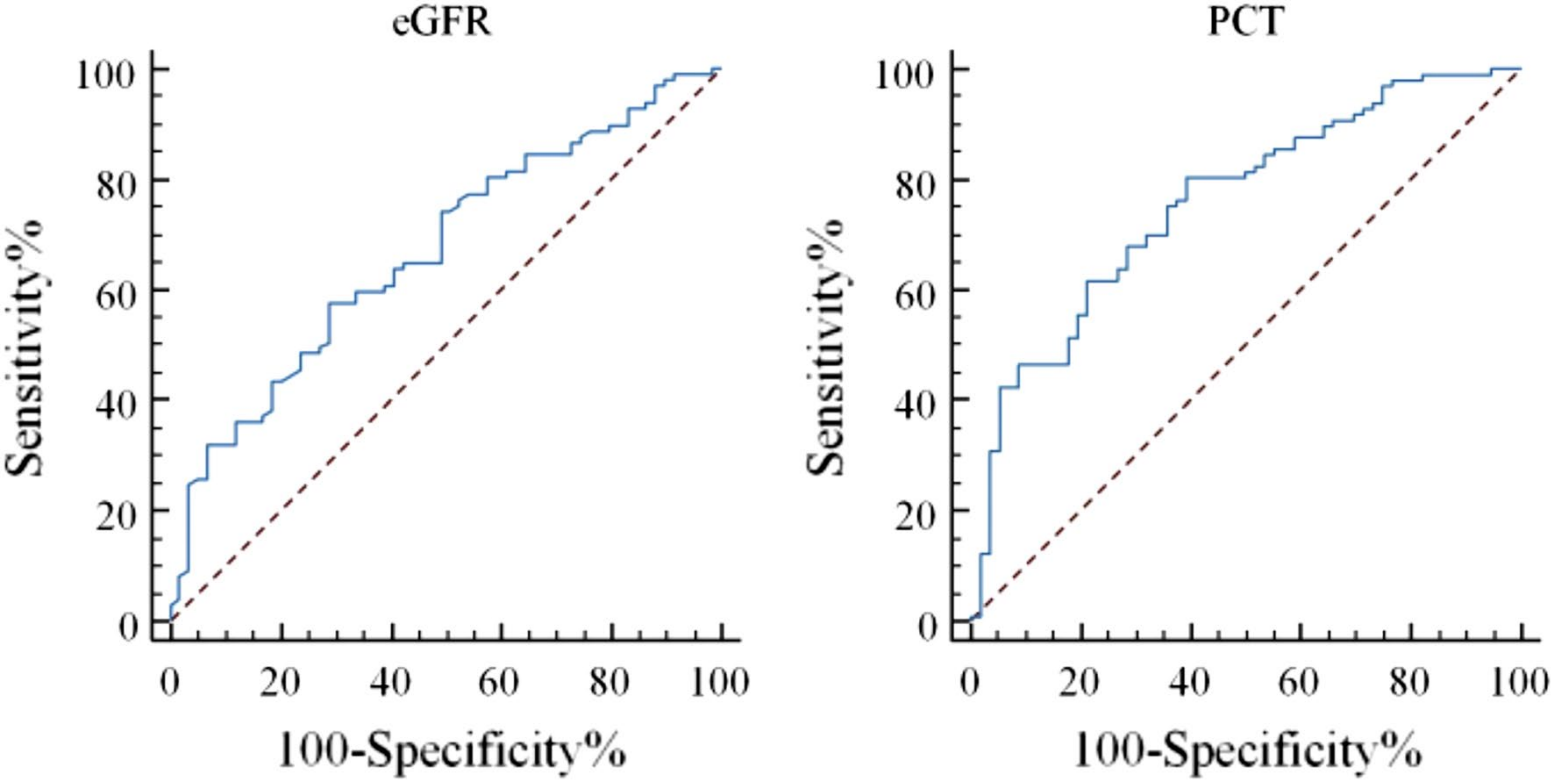
The area under the ROC curve of eGFR and PCT to predict emergency dialysis.

According to the results of multivariable logistic analysis, the continuous variables were selected for the receiver operating characteristic (ROC) curve. In this study, the area under the curve (AUC) of eGFR to predict emergency dialysis was 0.669 (95% CI 0.590–0.743), the sensitivity was 57.73%, the specificity was 71.19%, the best cut-off value was < 6.63 ml/min/1.73m^2^, and Youden's index was 0.289; while the AUC of PCT was 0.758 (95% CI 0.682–0.823), the sensitivity and specificity were 80.41% and 60.71%, respectively, the best cut-off value > 1.28 ng/ml, and Youden's index was 0.411 (Fig. 3).

## Discussion

RhBNP was associated with emergency dialysis and cardiovascular events. And eGFR and PCT were independent risk factors for emergency dialysis, and using rhBNP was a protective factor for emergency dialysis. This study suggest that rhBNP might improve cardiac function and reduce the occurrence of emergency dialysis and cardiovascular events in ESRD patients with type 4 CRS.

The increased burden of cardiovascular diseases in CKD patients and the influence of various chronic kidney impairments on the myocardium lead to the progression of heart damage. Cardiac manifestations include decreased heart function, myocardial remodeling, arrhythmias, and accelerated vascular calcification^17^. Some patients with type 4 CRS suffer from decompensated heart failure during the disease progression^4^. During the onset of heart failure, the decreased cardiac output leads to insufficient renal blood perfusion, and the increased sympathetic tension promotes renin secretion to activate the renin–angiotensin–aldosterone system (RAAS), further aggravating renal damage^18,19^. The above conditions form a vicious circle that precipitates the syndrome's deterioration. A considerable proportion of patients require premature kidney replacement therapy. Therefore, improving the therapeutic effect of type 4 CRS is vital.

BNP, mainly synthesized and secreted by ventricular myocytes, is a highly sensitive and specific indicator of heart failure^20^. This study evaluated the effect of rhBNP on emergency dialysis and prognosis in patients with CRS type 4 in end-stage renal disease ESRD. Compared with the non-rhBNP cohort, a significant improvement in daily urine output, BUN, IL-6, PCT, LVEF, PASP, LVDd, and LVDs, as well as lower sST2 and hsCRP levels, were seen in the rhBNP cohort. Multivariate regression analysis further showed that eGFR was a protective factor for emergency dialysis, so it is considered that residual renal function is still an important factor in delaying dialysis. Therefore, taking active measures to delay the aggravation of renal function is still the primary treatment in approaching type 4 CRS. This further suggests that RhBNP could improve cardiac function and reduce the occurrence of emergency dialysis and cardiovascular events in patients with type 4 CRS in ESRD.

NT-proBNP is another product of BNP precursor peptide cleaved by endonuclease that can be used as an important index for the diagnosis, evaluation, and prognosis of heart failure^21^. A cross-sectional study suggested that the diagnostic sensitivity and specificity of NT-proBNP are superior to those of BNP in patients with different CKD stages^22^. In this study, NT-proBNP levels of both cohorts decreased after treatment, but there were no significant differences in the decline between the two cohorts, which is considered for the improvement of patients' cardiac function and, subsequently, the rapidly dropped secretion of BNP after therapy resulting in insignificant differences between the two cohorts.

SST2, a novel biomarker for the diagnosis and prognosis of heart failure, is closely related to myocardial fibrosis, myocardial hypertrophy, and cardiac remodeling and is considered a strong, independent predictor of mortality and heart failure hospitalization in patients with acute or chronic heart failure^23^. Furthermore, it is considered more effective than natriuretic peptide in managing heart failure in patients with renal insufficiency as it is not affected by pre-existing cardiovascular disease, age, BMI, and renal function^24^. SST2 combined with natriuretic peptide can predict death, overall cardiovascular events, and heart failure^25^. Thus, the elevation of sST2 suggests poor prognosis in patients with heart failure^26^. In this study, we found that the decrease in sST2 level after therapy in the rhBNP cohort was greater than that in the non-rhBNP cohort, which suggested that the improvement of cardiac function was more obvious.

Due to the uremic toxins, volume overload, activation of the neuroendocrine system, oxidative stress, and other factors, most patients with CRS are in a state of inflammation, which promotes myocardial fibrosis and remodeling, thereby aggravating the deterioration of cardiac function^27^. Previous studies have shown that rhBNP can inhibit the inflammatory response, reduce the release of inflammatory cytokines and eliminate the immune responses induced by inflammatory cytokines^28^, alleviating inflammation damage to the myocardium. As a biomarker of inflammatory status in patients with CKD, hsCRP is also an important predictor of cardiovascular events, and its increase is associated with a higher incidence of cardiovascular events^29^. Our study found that the hsCRP level in the rhBNP cohort decreased more than that in the non-rhBNP cohort after treatment, suggesting that rhBNP ameliorates inflammatory state and reduces cardiovascular morbidity.

RhBNP can regulate reducing ventricular overload, depress pulmonary vascular resistance, and improve systolic cardiac function by its physiological effects of natriuresis/diuresis, peripheral vasodilatation and inhibiting the over-activation of the neuroendocrine system^30^. In this study, the increase in urine volume after treatment in the rhBNP cohort was larger than that in the non-rhBNP cohort, indicating rhBNP synergizes with traditional diuretics in enhancing diuresis. Also, the increase of LVEF and the decrease of PASP in the rhBNP cohort were greater than those in the non-rhBNP cohort, which was consistent with the existing literature^30^. Left ventricular remodeling is one of the most striking cardiac changes in patients with CKD, and the prevalence of left ventricular hypertrophy may gradually increase stepwise as the deterioration of GFR^4^. In the case of cardiac overload, the ventricular cavity compensatively expands in the early stage, and the compensatory mechanism dominated by the excitability of RAAS, sympathetic nervous system (SNS), and BNP can barely maintain normal cardiac output^31,32^. Yet, during this process, myocardial fibrosis is induced by the cytotoxicity of neurohumoral mechanisms after exceeding a certain limit. In this study, the decline of LVDs and LVDd was more remarkable in the rhBNP cohort, indicating that rhBNP effectively mitigates ventricular enlargement.

In recent years, a low proportion of ESRD patients received planned dialysis in China^33^. In contrast to planned approaches, urgent starts to dialysis are associated with higher mortality, medical complications, and costs^34^. In this research, the proportion of emergency dialysis in the rhBNP cohort was lower than that in the non-rhBNP cohort, and the difference was extremely significant, indicating that rhBNP might participate in preventing emergency dialysis, increasing the fraction of planned dialysis, improving the prognosis of patients and reducing the consumption of medical resources.

The incidence of cardiovascular events in patients with ESRD is approximately 10- 20 fold higher than that in the general population, and cardiovascular mortality accounts for 44%-51% of ESRD deaths^35^. Therefore, reducing cardiovascular morbidity performs fundamentally in the survival of patients with ESRD. In this study, the proportion of patients with short-term (one-month) cardiovascular events in the rhBNP cohort was lower than that in the non-rhBNP cohort, suggesting that rhBNP could improve short-term cardiac outcomes. Furthermore, longer follow-up of CRS patients treated with rhBNP in prior studies showed decreased cardiovascular events and lower readmission rates^36^. However, no significant differences were observed between the two cohorts in the proportion of patients undergoing cardiovascular events and maintenance dialysis within half a year, which may be due to the following: first, subjects in this study have progressed to ESRD, while patients in previous studies had relatively mild renal lesions; second, exogenous BNP supplementation could not improve the overall trend of disease; third, rhBNP cannot achieve long-term physiological effects due to its short half-life.

PCT is a precursor peptide of calcitonin that rapidly increases after bacterial infection, so it has been widely used for diagnosing infection and guiding antibiotics therapy. A recent study showed that procalcitonin could be used as a prognostic marker in heart failure with infection^37^, and in another study, low levels of elevated PCT were associated with increased mortality in patients with acute heart failure (AHF)^38^. Regarding kidney diseases, PCT has demonstrated optimal predictive ability for AKI in many clinical settings regardless of infection^39^. A case–control study reported that the PCT level in CKD patients was significantly higher than that in healthy controls and increased with advanced clinical stages^40^. We found that elevated PCT level is an independent risk factor for emergency dialysis in patients with type 4 CRS and ESRD in the absence of cases with severe infections in this retrospective study, suggesting that the increase in PCT is related to deteriorating cardiac and renal function. Therefore, we think it is important to explore PCT as a potential biomarker for the progression of cardiorenal syndrome.

The present study has several limitations. First, this was a single-center, retrospective study with a small sample size, possibly leading to a certain bias. Second, some qualitative data recorded by medical staff were not measured with a unified method, and there might be deviations in the frequency of visits due to individual differences in tolerance. Third, other drugs (such as diuretics and dopamine), cardiac function parameters, heart-related comorbidity, cardiac function grading, eGFR, urine volume, CKD parameters, etc., were not included because too many variables might affect test efficacy. Finally, patients' long-term prognoses were not adequately evaluated, given that rhBNP may not affect the patients’ conditions over a long period for the pharmacokinetic characteristics.

## Conclusions

RhBNP was associated with emergency dialysis and cardiovascular events. In addition, eGFR and PCT were independent risk factors for emergency dialysis, and using rhBNP was a protective factor. This study suggests that rhBNP might improve cardiac function and reduce the occurrence of emergency dialysis and cardiovascular events in ESRD patients with type 4 CRS. Yet, RCT studies with larger sample sizes and longer follow up are required to confirm these findings.

## Data Availability

Raw data used to support the findings of this study are available from the corresponding author upon request.
